# Female sex workers and police violence during the Covid-19 health crisis in 2020–2021: results from the EPIC multi-country community-based research program in Argentina

**DOI:** 10.1186/s12954-022-00714-5

**Published:** 2022-12-12

**Authors:** I. Aristegui, J. Castro Avila, V. Villes, R. M. Delabre, G. Orellano, M. Aguilera, M. Romero, L. Riegel, L. Kretzer, N. Cardozo, P. D. Radusky, D. Rojas Castro, EPIC Study Group

**Affiliations:** 1 Fundación Huésped, Research Department, Dr. Carlos Gianantonio 3932, C1202ABB Buenos Aires, Argentina; 2 Department of Research in Psychology, Universidad de Palermo, Buenos Aires, Argentina; 3 Community-Based Research Laboratory, Coalition PLUS, Pantin, France; 4 Asociación de Mujeres Meretrices de Argentina (AMMAR), Buenos Aires, Argentina; 5 Asociación de Travestis Transexuales y Transgénero de Argentina (ATTTA), Buenos Aires, Argentina; 6 Faculty of Psychology, Universidad de Buenos Aires, Buenos Aires, Argentina; 7 Inserm, IRD, SESSTIM, Sciences Economiques & Sociales de la Santé & Traitement de l’Information Médicale, ISSPAM, Aix Marseille Université, Marseille, France

**Keywords:** Female sex worker, Police violence, Covid-19, Argentina, Community-based organizations

## Abstract

**Background:**

Female sex workers (FSW) have been disproportionately impacted by the Covid-19 crisis. Data show increases of police violence toward key populations (KP), likely a consequence of their role in enforcing health government measures. This study aimed to identify factors associated with police violence experienced by FSW during the Covid-19 crisis in Argentina.

**Methods:**

EPIC is a multi-country, cross-sectional, community-based research program evaluating the impact of Covid-19 among KP. In Argentina, the study was conducted in collaboration with FSW community-based organizations (CBO). Participants completed an online survey (October 2020–April 2021). Police violence was measured as having experienced episodes of violence (physical, verbal, psychological or sexual) by security forces since the start of the health crisis. Factors associated with police violence were assessed in logistic regression models.

**Results:**

Among 173 respondents, median age was 34 [IQR 27–42], 39.3% were transgender women (TW), 78.1% declared sex work as their only income and 71.7% mentioned their financial situation has deteriorated with the health crisis. Nearly half of FSW (44.5%) reported experiencing police violence within the first year of the Covid-19 pandemic, and among them, 76.6% declared more frequent violence episodes since the beginning of the health crisis. After adjustment for age, being a TW (aOR [95% CI] = 2.71 [1.21;6.05]), reporting non-injection drug use (2.92 [1.02;8.36]), having a considerably deteriorated financial situation (3.67 [1.47;9.21]), having had a consultation with a CBO worker for medical care/treatments (5.56 [2.15;14.37]) and declaring fear or experiences of discrimination by physicians/other health workers (2.97 [1.21;7.29]), since the beginning of the Covid-19 health crisis, were independently associated with police violence.

**Conclusions:**

FSW in Argentina have experienced an increase in police violence since the beginning of the health crisis. Belonging to multiple KP (FSW, TW, people who use drugs) increases the likelihood of experiencing police violence, highlighting the need of an intersectional approach to develop interventions to reduce stigma and violence against FSW. CBOs have provided essential support and services during the crisis to FSWs, and other KPs, who may have avoided traditional healthcare structures due to fear or experiences of discrimination.

## Introduction

Female sex workers (FSW) have historically lived in a context of high psychosocial and economic vulnerability, associated with stigma and violence [1, 2]. Although sex work is not considered illegal in Argentina, some activities may be criminalized on the local level [1, 3]. Legal codes punishing sex work with arrests, fines or both, are still in force in 18 of the 24 districts of the country [4]. National and provincial laws and policies to fight human trafficking have indirectly and negatively affected autonomous sex work, by not making a distinction between forced and consented sex work and by fining and prosecuting sex work advertising in the street and communication media [1]. As shown elsewhere [5, 6], the lack of recognition of sex work as formal work contributes to a precarious economic situation, with high instability and difficulties to afford basic needs. Additionally, FSW experience high levels of stigma and discrimination related to sex work and violence from different sources, especially in the context of street-based sex work [7–9].

As FSW very often belong to other stigmatized groups, such as transgender women (TW), migrants and people living with HIV (PLHIV), among others, this may result in intersectional stigma, magnifying negative consequences on access to basic human rights. This adverse context in which FSW live and work greatly contributes to difficulties in access to healthcare, resulting in more physical and mental health problems [9, 10]. In Argentina, for example, HIV prevalence rates among FSW (2–3%) disproportionately exceeds that of the general population (0.4%) [11–13]. Moreover, among TW, a population with high engagement in sex work, HIV prevalence is estimated in 34% [11].

After the first cases of Covid-19 in Argentina, on March 20, 2020, the national government established a compulsory and highly restrictive lockdown that lasted until October 2020, when it was replaced by more flexible social distancing measures (e.g., use of masks in closed places and public transport) [14, 15]. The lockdown aimed to minimize face-to-face contact between individuals by restricting movement, work and travel to slow transmission and avoid overburdening of the healthcare system. Circulation was only allowed for those considered essential workers (e.g., healthcare workers) or for those with special permits for specific activities (e.g., attending a medical appointment) [14, 15]. Although measures implicated fines or arrest for non-complying people, these actions were rarely enforced. Additionally, international borders were closed. Most migrants were not able to return to their countries [14, 15]. Despite the efficacy of these measures to control the spread of Covid-19, they have had an overall negative economic impact, especially among groups that largely rely on informal economic activities to survive [10].

FSW have been disproportionately impacted by the Covid-19 health crisis, worsening their preexisting exposure to stigma and violence and their economic precarity [10, 16]. Local research showed that these measures have contributed to an aggravation of this psychosocial vulnerability and structural barriers to access basic human rights for FSW. Among 600 sex workers interviewed by AMMAR, the local sex workers’ organization, in the first 7 months of the pandemic, around 96% were female (cis and trans) and 72% were engaged in street-based sex work. Close to three quarters of the respondents (72%) described their personal economic situation as bad, with 96% reporting difficulties to afford basic needs such as food, clothes or shelter [17]. As reported internationally [10, 16], half of FSW experienced some kind of violence since the implementation of the lockdown and social distancing measures, with institutional violence being the most frequent [17]. Another study conducted during the first 2 months of the lockdown in Argentina showed that TW, a third of whom were engaged in sex work, reported negative socioeconomic changes, such as reduction in economic income and barriers to access basic services and goods (e.g., housing, food, hygiene products) [18]. Restrictions regarding circulation, for both sex workers and clients, and fear of contagion may have limited their opportunities to work. A consequence of this is loss of income and deterioration of the already precarious situation of FSW, increasing economic hardships and exclusion, as already reported in other contexts [5, 10, 16].

Furthermore, FSW have a long history of institutional violence and criminalization in Argentina. As previously stated, police can arrest or fine sex workers under some provincial codes, which may leave sex workers vulnerable to discretional and/or arbitrary police actions [1]. FSW working indoors (e.g., hotels, brothels, bars, massage parlors) experience these episodes during raids supported by current laws to prevent human trafficking, whereas other FSW are exposed to violence while working in the street. These episodes entail harassment, bribes (frequently, in the form of requests for free sexual services), stealing of FSW’s money or belongings and illegitimate and arbitrary searches and detentions, among others. A frequent justification for these actions is suspected drug trafficking or dealing [1]. This procedure is legitimized by the current legal framework about drugs in Argentina (Law N° 23,737), which criminalizes the use, possession and commercialization of any kind of illegal substance, including cannabis/marijuana and cocaine, the most frequently used [19].

Researchers and community organizations have warned that measures to control the Covid-19 health crisis may have exacerbated violence and discrimination against FSW and trans people, especially from the police and security forces [5, 16, 20]. Measures to control Covid-19 may have aggravated the preexisting situation by increased policing, which gave the police greater attributions to enforce compliance with lockdown and social distancing measures [16]. Due to loss of income and economic hardships, as well as insufficient financial assistance from the national government, FSW may have been forced to breach restrictions and social distancing measures in order to work and meet basic needs, increasing the odds of negative interactions, mistreatment, harassment and violence from the police, under the justification of non-compliance with lockdown restrictions.

In this context, researchers and sex workers’ organizations have made a call to action to protect sex workers during the Covid-19 health crisis and mitigate its adverse impact on this population [6, 21]. Community-based organizations (CBOs) have had an outstanding role in assisting and supporting their peers [5], rapidly developing strategies and resources to respond to the Covid-19 health crisis among sex workers [6, 16, 21]. Argentinian main sex workers’ CBOs implemented multiple actions, such as delivering food and hygiene products among FSW, assisting them in the obtention of circulation permits online, helping them apply to financial aids implemented by the national government, among other strategies [17]. However, these efforts should be complemented with evidence-based public policies that address this community’s needs and the structural factors that perpetuate their situation of vulnerability, including harassment and violence from security forces. Regarding this, it is noteworthy the scarcity of local evidence about factors associated with violence from the police during the Covid-19 health crisis and the impact of such violence on the FSW’s socioeconomic situation and access to healthcare. For this reason, the present study sought to identify factors associated with police violence experienced by FSW during the first year of the Covid-19 health crisis in Argentina.

## Methods

This study was conducted within the framework of the EPIC program, a multi-country, cross-sectional, and community-based study, conducted in more than 30 countries and coordinated by Coalition PLUS. The aim of this program was to assess the impact of the Covid-19 health crisis on populations vulnerable to, or living with HIV or HCV (Hepatitis C virus), and on people who work with these populations in community settings [22]. Briefly, the EPIC program was developed following information from CBOs members of Coalition PLUS about the concerning situation of key populations at the start of the Covid-19 health crisis and its eventual impact. After consultation with external scientific partners and representatives from the regional Coalition PLUS networks, a general mixed-methods community-based research protocol was developed. This protocol and associated study documents were specifically designed to be adapted to the local needs and contexts by the participating organizations. More precisely, for the quantitative surveys, participating organizations could choose among several modules based on the subject of interest (for example, sexual activity and prevention strategies for HIV and other STIs, experience and application of lockdown and/or barrier measures, access to healthcare) and target population (for example, sex workers, PLHIV, people who use drugs). Only one module, which included sociodemographic information and the general impact of Covid-19 health crisis, was mandatory. The study protocol and documents were developed collaboratively and made available to the Coalition PLUS network and scientific partners. The main study documents (protocol, questionnaires, including online questionnaire in Voxco®, interview guides, informed consent, etc.) were available in English, French, Portuguese or Spanish. In Argentina, the study exclusively focused on FSW.

### Participants

The sample consisted of FSW, both cis and trans, residing in Argentina since the outbreak of the Covid-19 health crisis. Inclusion criteria were to be 18 years old or older and to be engaged in sex work at the time of the survey or to have engaged in sex work prior to the start of the Covid-19 health crisis.

### Materials

The study questionnaire was constructed from the basis of the global EPIC program questionnaire, from which specific modules were selected and adapted based on local needs. In Argentina, the study was implemented as an online survey for FSW that was reviewed by members of two CBOs of FSW to better adapt wording to the community and also to incorporate questions that capture the specific situation of the Argentinian context. The final version consisted of two main blocks:

- The *sociodemographic component* explored age, gender identity (cis/trans), educational level, residency, housing conditions, identification with key populations (e.g., PLHIV, trans people, migrants, people who use drugs), financial changes due to the Covid-19 health crisis, perceived quality of life and impact of the Covid-19 health crisis on professional life. People who use non-injection drugs, for this study, are those who report identifying themselves as non-injection drug users, including illegal substances according to current Argentinian laws, and excluding prescription drugs, tobacco and alcohol. We also asked about injection drugs, but only three participants identified themselves with this group. Indeed, the most frequently used substances in Argentina are non-injectable (smoked, inhaled or snorted, such as cannabis/marijuana and cocaine).
- The block on *sex work* explored changes in sex work during the Covid-19 health crisis: importance of sex work as the source of income; changes in income since the start of the pandemic; changes in the experience of violence (physical, verbal, psychological and sexual) from security forces; fear or experiences of discrimination by health workers; and consultations with CBO workers to access medical care/treatments since the beginning of the Covid-19 health crisis. Police violence was asked using the following question: “Since the beginning of the Covid-19 health crisis, have you experienced episodes of violence by the police or security forces?”

### Study procedures

The study protocol, informed consent and other study documents were revised and approved by the Ethics Committee (Institutional Review Board) of Fundación Huésped. Prior to responding to the survey, participants were informed that their participation was voluntary and received information regarding the purpose of the study and the confidentiality of their responses before accepting the informed consent.

The data were collected in two phases. In the first phase (October–November 2020) participants were enrolled through outreach efforts by peer navigators involved in Fundación Huésped’s research team or working at the CBOs collaborating with this study. Flyers and other communication materials with the link to the survey were disseminated through institutional social media (e.g., Facebook, Instagram) of the CBOs, and through the peer navigators’ own contact list and close social media groups (e.g., WhatsApp). In the second phase (February–April 2021), collaborating CBOs implemented different strategies to reach members of the community who had expressed difficulties or barriers to respond to the online survey, but were willing to participate (e.g., limited access to Internet/Wi-Fi or electronic devices, difficulties in comprehension of the questions or difficulties in use of technology, among others). Peer navigators offered different forms of assistance in completing the survey: administering the survey as a structured interview (e.g., reading the questions to the participants and registering their answers) or staying close to the participant while responding to the survey in order to solve possible doubts or questions.

### Statistical analyses

Variables were compared between participants who have experienced episodes of police violence (physical, verbal, psychological or sexual) since the beginning of the Covid-19 health crisis, and those who have not experienced police violence since the beginning of the Covid-19 health crisis, using Chi-2 tests for categorical variables and Wilcoxon–Mann–Whitney tests for continuous variables. Logistic regression models were used to identify factors associated with “Having experienced episodes of police violence since the beginning of the Covid-19 health crisis” (yes/no). Variables with a *p*-value < 0.20 in the univariable analysis were considered eligible to enter the multivariable model. A backward procedure based on the Likelihood Ratio Chi-2 test was used to select significant variables for the final model (*p*-value < 0.05). Stata/SE 16.0 software [23] was used for all the analyses. Only variables with *p* < 0.05 in the multivariable model are shown in the respective table.

## Results

### Characteristics of the sample

Among 240 FSW in the EPIC Argentina study, 173 (72.1%) responded to the questions on police violence since the start of the Covid-19 health crisis (Table 1). Among them, the median age was 34 [IQR 27–42], 39.3% were TW and 69.9% were living in an urban area. Two out of three participants (66%) had been engaged in street-based sex work at some time. The majority (78.1%) reported that sex work was their only source of income and a similar proportion (71.7%) declared that their financial situation has deteriorated considerably since the health crisis. Nearly half of FSW (44.5%, *n* = 77) reported experiencing police violence since the beginning of the crisis and within the first year of the Covid-19 pandemic. Of them, 76.6% experienced more frequent violent episodes than before the Covid-19 health crisis.

**Table 1:** Descriptive statistics of sociodemographic characteristics and univariate models of factors associated with “Having experienced episodes of police violence” among FSW in Argentina (N = 173)

	Experiences of police violence		Total sample* N *= 173	*p*-value	Univariable *N* = 173	
	No *n* = 96 (55.5%)	Yes *n* = 77 (44.5%)				
	*n* (%)	*n* (%)	*N* (%)		OR [95% CI]	*p*-value
Age median [IQR] (for 1 year increase)	32 [25–40]	36 [29–44]	34 [27–42]	0.003	1.04 [1.01;1.08]	0.007
Gender identity				< 0.001		
Cis sex worker	74 (77.1)	31 (40.3)	105 (60.7)		1.00 [1.00;1.00]	
Trans sex worker	22 (22.9)	46 (59.7)	68 (39.3)		4.99 [2.58;9.64]	< 0.001
Current educational level				0.156		
Never went to school/ Primary education	33 (34.4)	33 (42.9)	66 (38.2)		1.00 [1.00;1.00]	
Secondary education	43 (44.8)	36 (46.8)	79 (45.7)		0.84 [0.43;1.61]	0.595
Higher education	20 (20.8)	8 (10.4)	28 (16.2)		0.40 [0.15;1.04]	0.059
Current place of residence				0.451		
Urban setting/big city	65 (67.7)	56 (72.7)	121 (69.9)		1.00 [1.00;1.00]	
Semi-urban environment/medium or small city	21 (21.9)	17 (22.1)	38 (22.0)		0.94 [0.45;1.95]	0.868
Rural setting/village	10 (10.4)	4 (5.2)	14 (8.1)		0.46 [0.14;1.56]	0.215
Current housing conditions				0.309		
Own home/rental home	77 (81.1)	66 (86.8)	143 (83.6)		1.00 [1.00;1.00]	
In a house hosted for free/						
Squatting/on the street/do not have a fixed home	18 (18.9)	10 (13.2)	28 (16.4)		0.65 [0.28;1.50]	0.312
Non-injection drug user				0.024		
No	82 (85.4)	55 (71.4)	137 (79.2)		1.00 [1.00;1.00]	
Yes	14 (14.6)	22 (28.6)	36 (20.8)		2.34 [1.10;4.97]	0.027
PLHIV				0.009		
No	89 (92.7)	61 (79.2)	150 (86.7)		1.00 [1.00;1.00]	
Yes	7 (7.3)	16 (20.8)	23 (13.3)		3.33 [1.29;8.59]	0.013
Migrant				0.004		
No	86 (89.6)	56 (72.7)	142 (82.1)		1.00 [1.00;1.00]	
Yes	10 (10.4)	21 (27.3)	31 (17.9)		3.22 [1.41;7.36]	0.005
Financial situation deteriorated considerably*				0.008		
No	35 (36.5)	14 (18.2)	49 (28.3)		1.00 [1.00;1.00]	
Yes	61 (63.5)	63 (81.8)	124 (71.7)		2.58 [1.27;5.27]	0.009
Worse quality of life*				0.047		
No	29 (30.2)	13 (17.1)	42 (24.4)		1.00 [1.00;1.00]	
Yes	67 (69.8)	63 (82.9)	130 (75.6)		2.10 [1.00;4.39]	0.049
Negative impact of the Covid-19 health crisis on the professional life in general				0.028		
No	19 (22.4)	7 (9.5)	26 (16.4)		1.00 [1.00;1.00]	
Yes	66 (77.6)	67 (90.5)	133 (83.6)		2.76 [1.09;6.99]	0.033
SW as currently main source of income				0.464		
No, I have another main activity	7 (7.5)	6 (7.9)	13 (7.7)		0.97 [0.31;3.03]	0.955
Yes, but I have other complementary activity	16 (17.2)	8 (10.5)	24 (14.2)		0.56 [0.23;1.41]	0.221
Yes, it is my only activity	70 (75.3)	62 (81.6)	132 (78.1)		1.00 [1.00;1.00]	
Decrease of the SW income received per week*				0.011		
No	12 (14.1)	2 (2.7)	14 (8.8)		1.00 [1.00;1.00]	
Yes	73 (85.9)	72 (97.3)	145 (91.2)		5.92 [1.28;27.4]	0.023
Fear or experiences of discrimination by physicians/other health workers*				0.044		
Never experienced this feeling	42 (46.1)	22 (29.7)	64 (38.8)		1.00 [1.00;1.00]	
Same as before/less often	25 (27.5)	20 (27.0)	45 (27.3)		1.53 [0.70;3.34]	0.289
More often	24 (26.4)	32 (43.3)	56 (33.9)		2.55 [1.22;5.33]	0.013
Consultation with a CBO worker for medical care/treatments				< 0.001		
No	79 (88.8)	39 (52.7)	118 (72.4)		1.00 [1.00;1.00]	
Yes	10 (11.2)	35 (47.3)	45 (27.6)		7.09 [3.18;15.79]	< 0.001

*Since the COVID-19 health crisis

### Univariate analyses

Compared to FSW who did not experience police violence since the beginning of the Covid-19 health crisis, those who experienced police violence were older (median [IQR]: 36 [29–44] vs. 32 [25–40] years, *p* = 0.003) and more often TW (59.2% vs. 22.9%, *p* < 0.001). They also were more frequently identified with the following key populations: people who use non-injection drugs (28.6% vs. 14.6%, *p* = 0.024), (20.8% vs. 7.3%, *p* = 0.009) and migrants (27.3% vs. 10.4%, *p* = 0.004). FSW who experienced police violence since the start of the Covid-19 health crisis, compared to FSW who did not, more often reported a deteriorated financial situation (81.8% vs. 63.5%, *p* = 0.008), a decrease in income/goods received per week from sex work (97.3% vs 85.9%, *p* = 0.011) and a negative impact of the Covid-19 health crisis on their professional life (90.5% vs. 77.6%, *p* = 0.028). Those who experienced police violence also declared a lower quality of life (82.9 vs. 69.8%, *p* = 0.047) and more often reported fear or experiences of discrimination by physicians/other health workers (43.3% vs. 26.4%, *p* = 0.044). Participants who reported police violence more often reported having had a consultation with a CBO worker for medical care/treatments during the health crisis (47.3% vs. 11.2%, *p* = 0.001).

### Multivariate analysis

A total of 163 (94.2%) FSW were included in the final multivariable model. After adjustment for age (Table 2), being a TW (aOR [95% CI] = 2.71 [1.21;6.05], *p* = 0.015), identifying as a person who uses non-injection drugs (2.92 [1.02;8.36], *p* = 0.046), having consulted with a CBO worker for medical care/treatments (5.56 [2.15;14.37], *p* < 0.001), having a considerably deteriorated financial situation (3.67 [1.47;9.21], *p* = 0.006) and having more often declared fear or experiences of discrimination by physicians/other healthcare workers (2.97 [1.21;7.29], *p* = 0.017), were independently associated with experiencing police violence since the start of the Covid-19 health crisis.

**Table 2:** Multivariate analyses of factors independently associated with “Having experienced episodes of police violence” among FSW in Argentina (*N *= 163)

	Multivariable model	
	aOR [95% CI]	*p-*value
Age (for one year increase)	1.04 [1.00;1.09]	0.042
Gender identity		
Cis sex worker	1.00 [1.00;1.00]	
Trans sex worker	2.71 [1.21;6.05]	0.015
Non-injection drug users		
No	1.00 [1.00;1.00]	
Yes	2.92 [1.02;8.36]	0.046
Financial situation deteriorated considerably*		
No	1.00 [1.00;1.00]	
Yes	3.67 [1.47;9.21]	0.006
Fear or experiences of discrimination by physicians/other health workers^*^		
Never experienced this feeling	1.00 [1.00;1.00]	
Same as before/less often	1.68 [0.63;4.52]	0.303
More often	2.97 [1.21;7.29]	0.017
Consultation with a CBO worker for medical care/treatments		
No	1.00 [1.00;1.00]	
Yes	5.56 [2.15;14.37]	< 0.001

*Since the COVID-19 health crisis

## Discussion

This study sought to identify factors associated with police violence experienced by FSW during the first year of the Covid-19 health crisis in Argentina. Almost half of the participants reported experiencing police violence in this period and most of them reported an increase in the frequency compared to before the crisis. These results confirm the warnings from researchers and sex workers’ organizations regarding an exacerbation of police violence and discrimination against this population during the Covid-19 health crisis [5, 16]. When governments worldwide increased policing to enforce Covid-19-related restrictions to circulation, this also involved greater attributions for the security forces to approach, interrogate and arrest citizens [5, 20]. As a result, these attributions may have provided security forces with greater agency to proceed against FSW, enhanced the preexisting criminalization of sex work activities in some areas and therefore increased the odds of police harassment and violence against this population, as results of this study show. Moreover, as streets and public places are the most frequent venues of sex work in Argentina [17] and considering that street-based sex work increases the odds of police violence [24], a reduction of people in the streets during lockdown may have left sex workers more unprotected when returning to the streets, as perpetrators of violence may have taken advantage of the lack of witnesses.

A considerable deterioration of the economic situation of FSW was independently associated with police violence. Participants that experienced police violence were also more likely to report a reduction of their weekly income since the beginning of the Covid-19 health crisis, which has also had a negative impact on their quality of life. In accordance with reports from international and local researchers and sex workers’ organizations [10, 16, 17], this study found a general worsening of the already precarious working conditions and socioeconomic situation among FSW in Argentina, probably owing to sex work not being recognized as a formal activity and still being criminalized in some areas of the country [1, 4]. This situation deteriorated even more after hotels, brothels, bars, massage parlors and other venues were forced to close during lockdown, as they were categorized as non-essential. This measure may have legitimized raids that frequently involved violence against FSW who tried to continue working indoors in these venues. Moreover, without these venues, some FSW may have been forced to provide their services in public places and/or rely on street-based sex work as the only option for livelihood. As circulation in streets was restricted unless a special permit was granted, security forces had additional latitude for raiding traditional sex work areas. The lack of special permits to circulate may have been used as a reason for detention, leading to greater exposure to harassment. This situation may also have contributed to a reduction in the number of clients, as they were subject to the same circulation restrictions and could be questioned and detained by the police. In sum, sex work was strongly limited or even impossible to practice without being exposed to police actions and retaliation.

Police violence was also independently associated with being a TW, and a person who uses non-injection drugs. These findings are consistent with reports that have shown that transgender and undocumented FSW present a greater vulnerability to punitive Covid-19 control measures [25, 26]. TW have a long history of violence and harassment from the police that is related to their gender identity [27]. In Argentina, several studies have also documented considerable levels of police violence and discrimination against this population [28–30]. Furthermore, TW from Buenos Aires have identified the streets and police stations as the most violent settings [29] for their community. Several researchers have warned that police violence against transgender people was exacerbated during the Covid-19 health crisis [18, 20].

The role of non-injection drugs use was highlighted in this study in relation to police violence. An association between substance use and police harassment among FSW has been already reported, especially in the context of street-based sex work [24, 31]. International evidence consistently shows high prevalence of substance use among FSW, even during the Covid-19 pandemic [24, 32, 33]. In Argentina, evidence is scarce, though elevated substance use was found in a sample of TW, most of whom were engaged in sex work [30]. In particular, during lockdown, FSW who use substances may have increased their exposure to police violence when going out to acquire them. It is also possible that some FSW may have been forced to engage in small-scale drug dealing as a source of income. Regardless, suspicion of drug possession for personal use or for commercialization is a reason commonly argued by the police to approach, interrogate and incriminate FSW, even in violent or abusive ways [1, 31].

Belonging to other stigmatized groups, such as being migrants or PLHIV, was also associated with police violence. Migrant FSW may experience several factors that contribute to a more precarious situation. They may possibly not be acquainted with the local laws (including their rights), they may lack local identity documents or may not have permanent residency in the country. Therefore, they are at a greater disadvantage to defend themselves against police harassment, violence and abuse. This has already been reported in other contexts [5, 6] and locally [17]. Additionally, they may not be aware of the available government resources for self-defense, where to turn to ask for help, or lack family networks that assist them. Similarly, FSW living with HIV, who had to leave their homes to attend medical appointments and pick up antiretroviral medicine, may have been more exposed to police interrogation. Even though circulation permits were granted for medical reasons, the police were authorized to request them at any time. Previous traumatic experiences with the police may have influenced the interactions between FSW and police officers even out of the context of sex work.

FSW who reported police violence during the Covid-19 health crisis were more likely to have declared an increase in the frequency of fear or experiences of discrimination by physicians or other healthcare workers since the beginning of the Covid-19 health crisis. Previous research has shown that stigma related to sex work and discrimination from physicians and healthcare workers have traditionally been a barrier to healthcare access in this population and a recurrent reason to avoid attending healthcare services, even before the Covid-19 health crisis [7–9, 34]. Moreover, experiences of physical and sexual violence have been found associated with fear of seeking healthcare services among FSW [31]. Although difficult to explain, the association between police violence and access to healthcare has been found at different levels [3]. Allam et al. [35] showed that having been arrested in the last year was related to non-adherence to HIV treatment among FSW. Similarly, local pre-pandemic evidence showed an association between gender identity-related police discrimination and low adherence to HIV care among TW from Buenos Aires, Argentina; many of whom were engaged in sex work [36].

There may be several explanations for this result. Firstly, the institutional violence involved in the abuse and harassment from the police may be generalized to other institutions. Therefore, FSW anticipate stigma and discrimination in different institutional settings, such as healthcare services, especially if there is a history of mistreatment from physicians and other healthcare workers. Secondly, FSW who experienced police violence may have developed chronic fear and anticipation of discrimination of uniformed personnel, including security staff and guards, which are frequently the first staff they would meet when attending clinics and hospitals.

In contrast, FSW who reported experience of police violence were more likely to have had a consultation with a community worker to access healthcare. As reported worldwide [37], these results highlight the role of community workers and CBOs in assisting FSW and facilitating access to healthcare during the Covid-19 health crisis. Community workers are frequently peers, have a better understanding and awareness of FSW’s adverse situations and thus may be more effective addressing their needs [13, 31]. They also grant a safe, accepting environment and a stigma-free interaction for FSW. In this sense, it is possible that FSW who experienced police violence also experienced higher vulnerability and were more likely to turn to CBOs and their peers for assistance and help. A local study has described how peer navigators facilitate access to healthcare and other kinds of assistance among the community of TW from Buenos Aires, who show high proportions of engagement in sex work. Peer navigators function as a bridge between the community and the healthcare services [38]. This role may have become even more fundamental during the Covid-19 health crisis, as barriers to access healthcare have been amplified for FSW for all the reasons stated above [18, 25]. Therefore, CBOs and community workers may have been the first option for assistance and protection in situations of violence and may have compensated for the barriers to access medical attention and treatment since the start of the pandemic.

Furthermore, several researchers and community leaders have highlighted how sex workers' organizations have developed rapid responses to meet the community’s needs and support their peers during the Covid-19 health crisis, showing high levels of resilience. This included strategies to support and protect sex workers experiencing violence and advocacy to end state-promoted policing [5, 17, 25]. In line with previous studies [31], these results highlight the need for intervention programs in violence prevention and risk reduction strategies among FSW. Additionally, considering the pivotal role of CBOs during the pandemic, the collaborative development of health interventions is necessary for greater impact.

Some limitations of this study can be mentioned. Firstly, a small non-probability sample was enrolled. As it may not be representative of the rest of the population of FSW from Argentina, generalizability of these results is limited. Sample size may also have limited statistical power analysis. Secondly, information was collected through an online survey, which may imply several biases. Self-report may involve social desirability bias, whereas the online procedures may be more easily available to some participants than to others, depending on access to electronic devices and familiarity with their use. Procedures were implemented to reduce this last bias (e.g., administration of the survey as an interview by peer navigators/promoters). However, these options were only limited to FSW that could be reached by peer navigators, and for some FSW groups or communities the only option available was online. Enrollment of participants and data collection were implemented in two phases that involved different strategies. This may have also introduced bias in the results and should be acknowledged. Thirdly, given the cross-sectional nature of the study, causality or direction of some of the observed associations with police violence cannot be inferred. Finally, due to the nature of the question or the distribution of the sample, the role of some variables, such as effective access to healthcare or the type of sex work venues, was not possible to be analyzed. Also, social class status, another possible reason for intersectional discrimination against FSW, could not be explored in this study and future research should consider including and analyzing the role of this variable.

Future studies would benefit from achieving a nation-wide sample, analyzing the role of different sex work venues in relation to violence and exploring the relationship between police violence and healthcare-seeking behaviors. This information is critical to accurately inform programs and public policies aiming to improve FSW’s quality of life and healthcare access, and to prevent police violence.

## Conclusion

Despite its limitations, this study provides evidence on the impact of the Covid-19 health crisis on this vulnerable population, and it is one of the first research studies that is specifically focused on the role of police violence among FSW in Argentina. The results from the EPIC community-based research program show that police violence against FSW in Argentina increased during the Covid-19 health crisis. Having experienced police violence since the beginning of the Covid-19 health crisis was associated with identifying as a person who uses non-injection drugs, being a TW, having a deteriorated financial situation, declaring fear or discrimination in healthcare settings and having had a consultation with a CBO worker for medical care. Particularly, this research highlights the role of intersecting stigmatized identities (including using non-injection drugs) in exposure to police violence, but also showed the resilient role of the FSW community-led organizations in facilitating access to medical services during the Covid-19 health crisis. Collaborations between research institutions and the community of FSW, as in the development and conduct of this study, may be pivotal in the design of effective strategies aiming to reduce police violence and its detrimental effect on this vulnerable population.

## Data Availability

The datasets used and/or analyzed during the current study are available from the corresponding author on reasonable request.
